# Tid1-S regulates the mitochondrial localization of EGFR in non-small cell lung carcinoma

**DOI:** 10.1038/oncsis.2017.62

**Published:** 2017-07-17

**Authors:** T-H Wang, Y-H Lin, S-C Yang, P-C Chang, T-CV Wang, C-Y Chen

**Affiliations:** 1 Graduate Institute of Health Industry Technology and Research Center for Industry of Human Ecology, College of Human Ecology, Chang Gung University of Science and Technology, Tao-Yuan, Taiwan; 2 Tissue Bank, Chang Gung Memorial Hospital, Tao-Yuan, Taiwan; 3 Institute of Biomedical Sciences, Academia Sinica, Taipei, Taiwan; 4 Department of Molecular and Cellular Biology, College of Medicine, Chang Gung University, Tao-Yuan, Taiwan

## Abstract

The epidermal growth factor receptor (EGFR) is the major driver of non-small cell lung carcinoma (NSCLC). Mitochondrial accumulation of EGFR has been shown to promote metastasis in NSCLC, yet little is known about how the mitochondrial localization of EGFR is regulated. In this work, we show that Tid1 (also known as mitochondrial HSP40) is involved in the mitochondrial localization of EGFR, and that the DnaJ domain of Tid1-S is essential for the Tid1-S-mediated transportation of EGFR into mitochondria. Overexpression of Tid1-S increased the migration and invasion of NSCLC cells cultured *in vitro*. High levels of EGFR and Tid1-S were detected in the mitochondria of cancerous lesions from stage IV NSCLC patients, and high levels of mitochondrial Tid1-S/EGFR were correlated with lymph node metastasis and poor overall survival of NSCLC patients. We thus conclude that Tid1-S critically governs the mitochondrial localization of EGFR through the mtHSP70 transportation pathway, and that the mitochondrial accumulation of EGFR appears to promote metastasis in NSCLC.

## Introduction

Lung cancer is the leading cause of cancer death worldwide, and non-small cell lung cancer (NSCLC) is the dominant type of this disease.^1^ Epidermal growth factor receptor (EGFR) is a receptor tyrosine kinase (RTK) that acts as the major driver of NSCLC; it regulates important tumorigenic processes, including proliferation, apoptosis, angiogenesis, invasion and drug resistance.^2^ EGFR is well known to localize to the plasma membrane, where it phosphorylates downstream substrates on their tyrosine residues in response to extracellular stimulation. Activated EGFR normally undergoes endocytosis and subsequent transportation to various organelles for degradation, recycling, or to perform functions that remain poorly understood.^3^ Recent studies showed that EGFR can translocate into the nucleus and mitochondria,^3^ but although nuclear EGFR is thought to function as a transcription factor,^4^ relatively little is known about the translocation/function of mitochondrial EGFR. We do know that EGF stimulation enhances the mitochondrial localization of EGFR and decreases cellular ATP in breast cancer cells,^5^ and that the mitochondrial translocation of EGFR enhances NSCLC cancer cell invasion and metastasis by regulating mitochondrial dynamics.^6^ Moreover, ErbB2, another member of the EGFR protein family, reportedly translocated into mitochondria and regulates cellular metabolism in breast cancer.^7^ These studies suggest that the localization of EGFR to mitochondria can affect cellular metabolism related to tumorigenesis. To date, however, little is known about how the mitochondrial localization of EGFR is regulated.

Tid1, which is also known as mitochondrial heat shock protein 40 (mtHSP40), contains a conserved DnaJ domain through which it interacts with members of the heat shock protein 70 (HSP70) family of chaperone proteins.^8^ Two alternatively spliced isoforms, Tid1 long form (Tid1-L) and Tid1 short form (Tid1-S), are expressed in human cells.^9^ The N-terminus of Tid1 bears a mitochondrial signal sequence, and a major fraction of endogenous or ectopically expressed Tid1 was found to reside in the mitochondrial matrix.^10^ Tid1-L has a greater cytosolic stability and a lower rate of mitochondrial import compared with Tid1-S, meaning that Tid1-S predominates in the mitochondria.^11^ Tid1 has been shown to form a complex with p53 under hypoxic conditions; this complex directs the mitochondrial translocation of p53 and the subsequent initiation of the mitochondrial apoptosis pathway in breast cells.^12^ Mitochondrial Tid1 has been shown to interact with the amino-terminal segment of adenomatous polyposis cell (APC) tumor suppressor to facilitate apoptosis.^13^ Therefore, Tid1 appears to participate in transporting candidate proteins to mitochondria and regulating the function of mitochondrial proteins.

Several studies have indicated that Tid1 is involved in regulating RTK-mediated signaling, such as through EGFR, ErbB2 and cMet.^14, 15, 16^ Our group has recently shown that Tid1-L can interact with EGFR through the HSP90/HSP70 complex, and that it inhibits EGFR signaling by enhancing the ubiquitinylation and degradation of EGFR.^17^ However, it is not yet known whether Tid1 can participate in the mitochondrial translocation of EGFR and/or regulate its functions in mitochondria. In this work, we examined the role of Tid1 in the mitochondrial localization of EGFR in NSCLC.

## Results

### Tid1-S is involved in the mitochondrial translocation of EGFR in NSCLC cells

**Figure 1:**
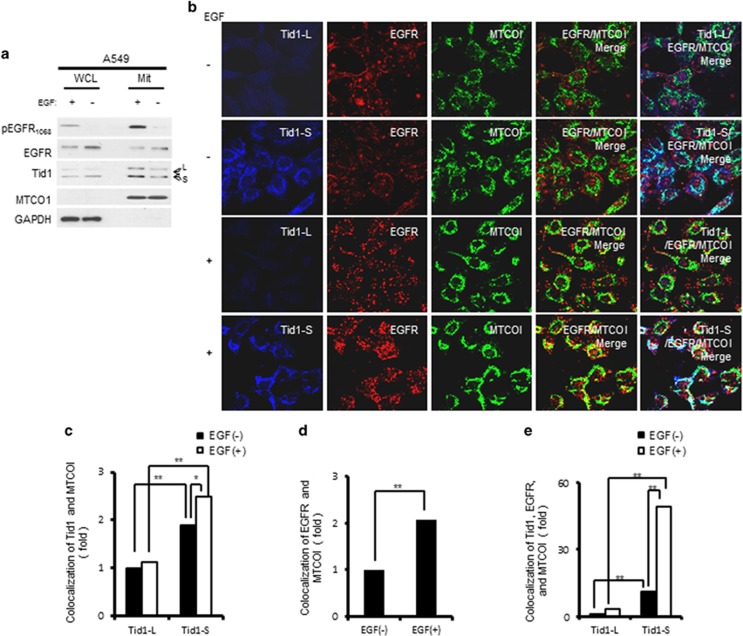
Effect of EGF stimulation on the mitochondrial translocation of EGFR. A549 cells were serum starved, treated with 50 ng/ml of EGF for 15 min, and then subjected to mitochondrial fractionation. (**a**) Western blot analyses of relevant protein levels in mitochondrial fractions (Mit, 10 μg) and the remaining portion of whole-cell lysates (WCL, 20 μg). The cytoplasmic GAPDH and mitochondrial MTCO1 markers were included to validate the purity of the mitochondrial fraction. The endogenous long form (L) and short form (S) of Tid1 are indicated by arrows marked L and S, respectively. The relative amounts of proteins present in the gel were quantified by densitometry. (**b**) Immunofluorescence (IF) staining for the subcellular distributions of Tid1-S, Tid1-L, EGFR and MTCOI. The colocalization of EGFR and MTCO1 in mitochondria could be visualized as yellow-colored spots in the EGFR/MTCO1 Merge. The colocalization of Tid1 (-L or -S) and EGFR in mitochondria could be visualized as white-colored spots in the Tid1/EGFR/MTCO1 Merge. (**c-e**) The colocalizations of Tid1 and MTCOI (**c**), EGFR and MTCOI (**d**), or Tid1, EGFR, and MTCOI (**e**) in~200 cells per experiment were scored and analyzed using the MetaMorph software. The scores are expressed as a fold-increase over the corresponding colocalization in untreated cells (-EGF), which was set as 1. The data shown in C-E represent the means±SD from three independent experiments; *P<0.05, and **P<0.01, as assessed using the Student’s *t*-test.

As EGF stimulation has been shown to enhance the mitochondrial localization of EGFR, we examined whether Tid1 appeared to be involved in this process. A549 cells were serum starved for 24 h and then treated with EGF for 15 min, and the translocation of EGFR to mitochondria was analyzed. As shown in Figure 1a, EGFR was phosphorylated (activated) upon EGF stimulation. Moreover, the phosphorylated EGFR was readily detected in mitochondria, suggesting that some of the activated EGFR was transported to the mitochondria. Based on the amounts of extracted proteins and the amounts of proteins loaded in the gel for analysis, we estimated that about 2% of phosphorylated EGFR was transported to the mitochondria. However, analysis of the ratios of total EGFR in mitochondria revealed that the fractions of mitochondrial EGFR before and after EGF stimulation were not significantly changed and stayed at 0.02. To evaluate whether Tid1 was involved in the mitochondrial translocation of EGFR, we used immunofluorescence (IF) staining to analyze the subcellular distributions of EGFR, Tid1-L, Tid1-S and MTCOI (a mitochondrial marker). As shown in Figure 1b, in the absence of EGF stimulation, mitochondria contained little EGFR, but harbored most of the expressed Tid1-L and Tid1-S. Upon EGF stimulation, the colocalization of Tid1-S and EGFR with MTCO1 increased, whereas there was little change in Tid1-L (Figures 1c and d). Importantly, the three-way colocalization of EGFR, Tid1-S, and MTCO1 was dramatically increased upon EGF stimulation (Figure 1e), suggesting that Tid1-S is involved in the EGF-stimulated translocation of EGFR into mitochondria.

**Figure 2:**
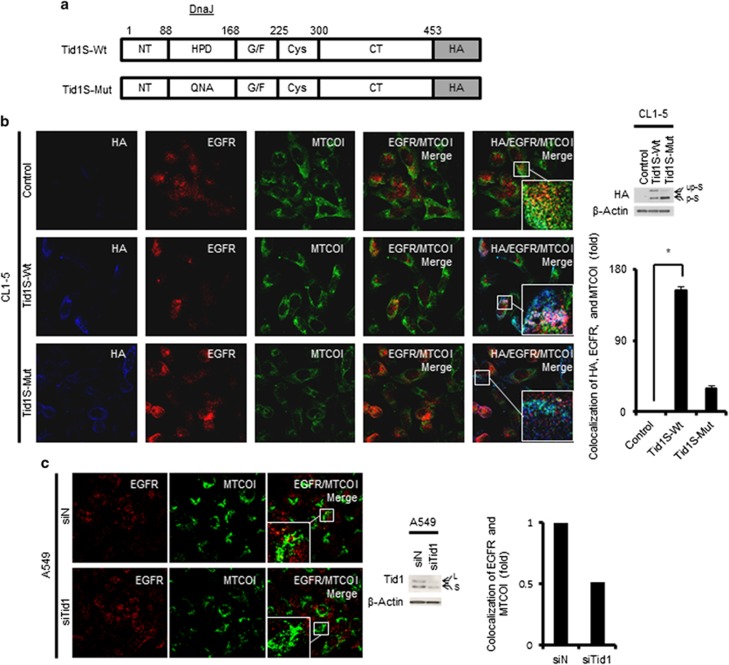
Effects of Tid1-S overexpression or knockdown on the translocation of EGFR into mitochondria. (**a**) Schematic illustration of wild-type Tid1-S-HA (Tid1-S-Wt) and the DnaJ domain mutant Tid1-S-HA (Tid1-S-Mut), in which the amino acid sequence H121P122D123 (wild-type) was replaced with Q121N122A123 (mutant) in the DnaJ domain of Tid1-S. (**b**) CL1-5 cells were transfected with Tid1-S-Wt, Tid1-S-Mut, or empty vector (control) and cultured for 24 h. Representative IF staining with anti-HA, -EGFR and -MTCOI antibodies is shown in the left panel. The colocalization of EGFR and MTCO1 in mitochondria could be visualized as yellow-color in the EGFR/MTCO1 Merge. The colocalization of HA, EGFR, and MTCOI in mitochondria was detected as white color in the HA/EGFR/MTCO1 Merge. The expression of HA-tagged Tid1-S-Wt or Tid1-S-Mut in the transfected CL1-5 cells was determined by Western blotting, and is shown in the right-upper panel. The arrows indicated by ‘up-S’ and ‘p-S’ show the positions of the unprocessed form (up) and the processed form (p) of Tid1-S-HA (S), respectively. β-Actin was used as a loading control. The colocalization of HA-Tid1-S, EGFR and MTCOI in~200 cells was analyzed using the MetaMorph software. The scores are expressed as a fold-increase over that of the vector-transfected control cells, which was set as 1. The data shown in the right-lower panel were from three independent experiments. (**c**) A549 cells were transfected with siTid1 or siN for 72 h. Representative IF staining with anti-EGFR and anti-MTCOI antibodies is shown in the left panel. The expression level of Tid1 in the transfected cells was analyzed by Western blotting, and is shown in the middle panel. β-Actin was used as a loading control. The scores for the colocalization of EGFR and MTCOI in~200 cells was analyzed using the MetaMorph software (right panel). The data shown are the means±SD from three independent experiments; *P<0.05, as assessed with the Student’s *t*-test.

To confirm the role of Tid1-S in the mitochondrial localization of EGFR, we examined the effect of Tid1-S overexpression on the mitochondrial localization of EGFR in a human lung cancer cell line that expresses a low level of endogenous Tid1 (CL1-5 cells).^17^ Overexpression of HA-tagged wild-type Tid1-S (Tid1-S-Wt) (Figure 2a) greatly enhanced the co-localization of EGFR with Tid1-S in mitochondria (Figure 2b). Conversely, the siRNA-mediated depletion of endogenous Tid1 from A549 cells decreased the mitochondrial localization of EGFR (Figure 2c). Together, these data support the hypothesis that Tid1-S is involved in the mitochondrial accumulation of EGFR in NSCLC.

### The DnaJ domain of Tid1-S is essential for the interaction with and mitochondrial localization of EGFR

**Figure 3:**
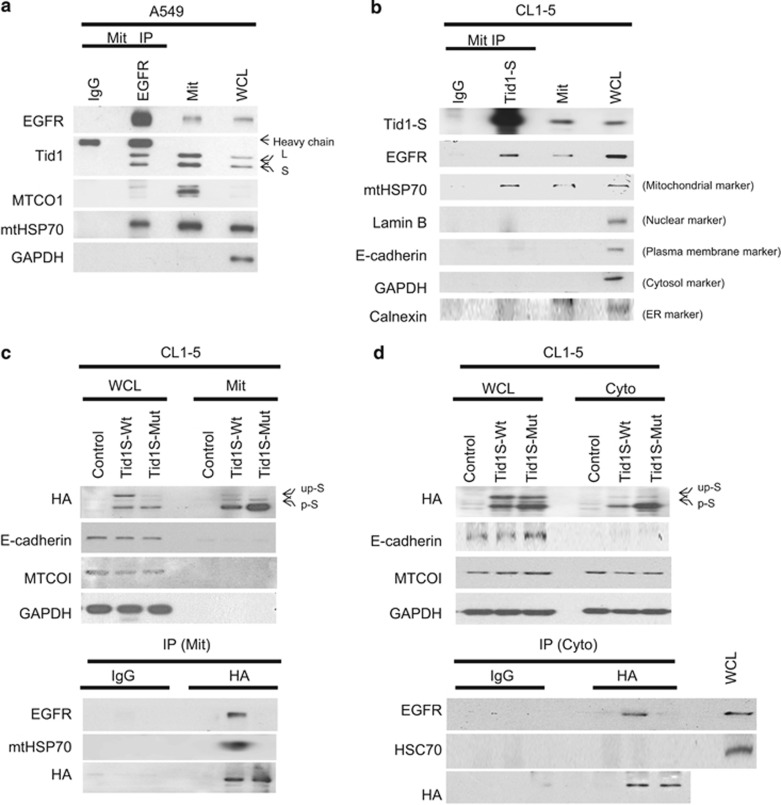
Tid1-S interacts with EGFR and HSP70 in mitochondria. Cell lysates from A549 (**a**) and CL1-5 (**b**) cells were subjected to mitochondrial fractionation. The proteins from the mitochondrial fraction (Mit, 1 mg) were immunoprecipitated with anti-EGFR (**a**) or anti-Tid1-S (**b**), and the proteins in the immunoprecipitates were analyzed by western blotting. The utilized markers were as follows: MTCO1 and mtHSP70 for mitochondria; Lamin B for nuclei; E-cadherin for plasma membrane; and GAPDH for cytoplasm. Endogenous Tid1-L and Tid1-S are indicated by arrows marked ‘L’ and ‘S,’ respectively. (**c**, **d**) CL1-5 cells were transfected with HA-Tid1-S-Wt, HA-Tid1-S-Mut, or empty vector. The cells were cultured for 24 h, and cell extracts were subjected to mitochondrial fractionation. Proteins from the mitochondrial fraction (Mit, 1 mg; (**c**) and cytosolic fraction (Cyto, 1 mg; (**d**) were immunoprecipitated with anti-HA-conjugated agarose beads, and the immunoprecipitated proteins were analyzed by western blotting. The ‘up-S’ and ‘p-S’ indicate the positions of the unprocessed form (up) and the processed form (p) of Tid1-S-HA (S), respectively.

To explore the function of mitochondrial EGFR, we sought to identify its interacting partners in mitochondria. Mitochondrial proteins were obtained from A549 cells and immunoprecipitated with anti-EGFR or anti-Tid1-S antibody. As shown in Figure 3a, mitochondrial HSP70 (mtHSP70) and Tid1 (both Tid1-L and Tid1-S) were detected in the immunoprecipitates. In a reciprocal immunoprecipitation study with anti-Tid1-S antibody, EGFR and mtHSP70 were detected in the immunoprecipitates (Supplementary Figure 1). Similarly, when the mitochondrial proteins from CL1-5 cells were immunoprecipitated with an anti-Tid1-S antibody, EGFR and mitochondrial HSP70 (mtHSP70) were readily detected in the immunoprecipitates (Figure 3b). Thus, Tid1-S appears to interact with mtHSP70 and EGFR.

As the DnaJ domain of Tid1 contains a HPD motif (tripeptide of histidine, proline and aspartic acid) that is known to interact with mtHSP70 but not cytosolic HSP70 (HSC70),^10, 18^ we speculated that the observed interaction of Tid1-S with mtHSP70 and EGFR could be mediated through the DnaJ domain. To investigate the role of the DnaJ domain in the Tid1-S-mediated mitochondrial accumulation of EGFR, we replaced the HPD (amino acids 121–123) of Tid1 with QNA to generate a Tid1-S triple mutant (Tid1-S-Mut) (Figure 2a). We transfected plasmids encoding HA-tagged wild-type Tid1-S (Tid1-S-Wt) or the HA-tagged DnaJ domain mutant of Tid1-S (Tid1-S-Mut; Figure 2a) into CL1-5 cells, examined the co-localization of EGFR and Tid1-S in mitochondria, and assessed the interactions of Tid1-S with EGFR and mtHSP70. As shown in Figure 3c, both EGFR and mtHSP70 were readily detected in the mitochondrial anti-HA immunoprecipitates of cells transfected with Tid1-S-Wt but not Tid1-S-Mut. HSC70 was not detected in the cytosolic anti-HA immunoprecipitates of cells transfected with Tid1-S-Wt or Tid1-S-Mut. EGFR was only detected in the cytosolic anti-HA immunoprecipitates from cells transfected with Tid1-S-Wt (Figure 3d). The apparent specific interaction of Tid1-S with mtHSP70 suggests that Tid1-S may serve a critical function in the mtHSP70 chaperone system. Moreover, our results indicate that the DnaJ domain of Tid1-S is indispensable for the Tid1-S-mediated transportation of mitochondrial EGFR.

### Overexpression of Tid1-S increases the migration and invasion of cultured NSCLC cells

**Figure 4:**
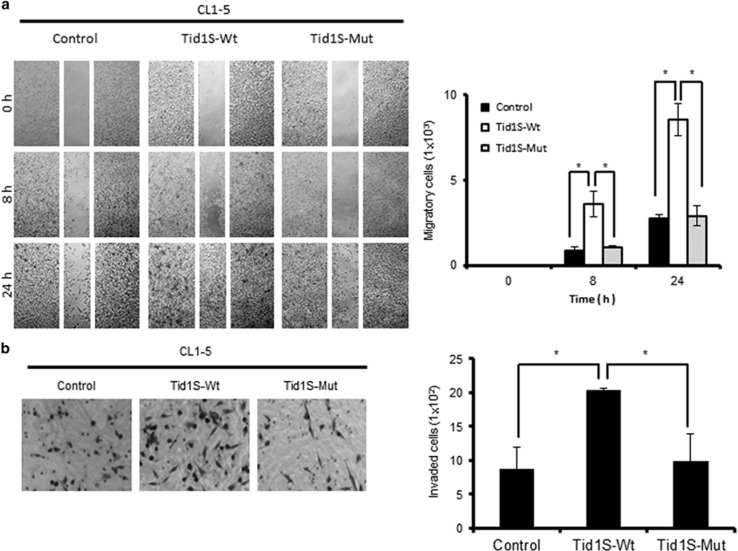
Effect of Tid1-S overexpression on the migration and invasion of CL1-5 cells. CL1-5 cells were transfected with Tid1-S-Wt, Tid1-S-Mut, or empty vector and cultured for 48 h. The transfected cells were examined for their migration ability in a wound-healing assay (**a**) and for their invasion ability in a Matrigel assay (**b**). Representative results are shown in the left panels. The data shown in the right panels are from three independent experiments performed on triplicate samples; *P<0.05, as assessed with the Student’s *t*-test.

Mitochondrial translocation of EGFR has been shown to promote metastasis in NSCLC.^6^ Our finding that Tid1-S is involved in the mitochondrial translocation of EGFR prompted us to question whether Tid1-S could contribute to regulating metastasis in NSCLC. To assess this hypothesis, we examined the effect of Tid1-S overexpression on cell migration and invasion *in vitro*. CL1-5 cells were transfected with HA-tagged Tid1-S-Wt, HA-tagged Tid1-S-Mut, or vector alone for 24 h, and the cells were assessed for their migration and invasion abilities. As shown in Figure 4a, Tid1-S-Wt-expressing CL1-5 cells showed significantly increased motility in a wound-healing assay, compared with CL1-5 cells transfected with Tid1-S-Mut or the control vector. Similarly, a Matrigel invasion assay showed that the invasion ability of Tid1-S-Wt-expressing CL1-5 cells was significantly higher than those of CL1-5 cells transfected with Tid1-S-Mut or the control vector (Figure 4b). A similar effect of Tid1-S overexpression on the migration and invasion was also observed in A549 cells (Supplementary Figure 2). Together, these results suggest that Tid1-S has an important function in modulating cell migration and invasion in NSCLC cells.

### High mitochondrial levels of Tid1-S/EGFR correlate with lymph node metastasis and poor overall survival in patients with NSCLC

**Figure 5:**
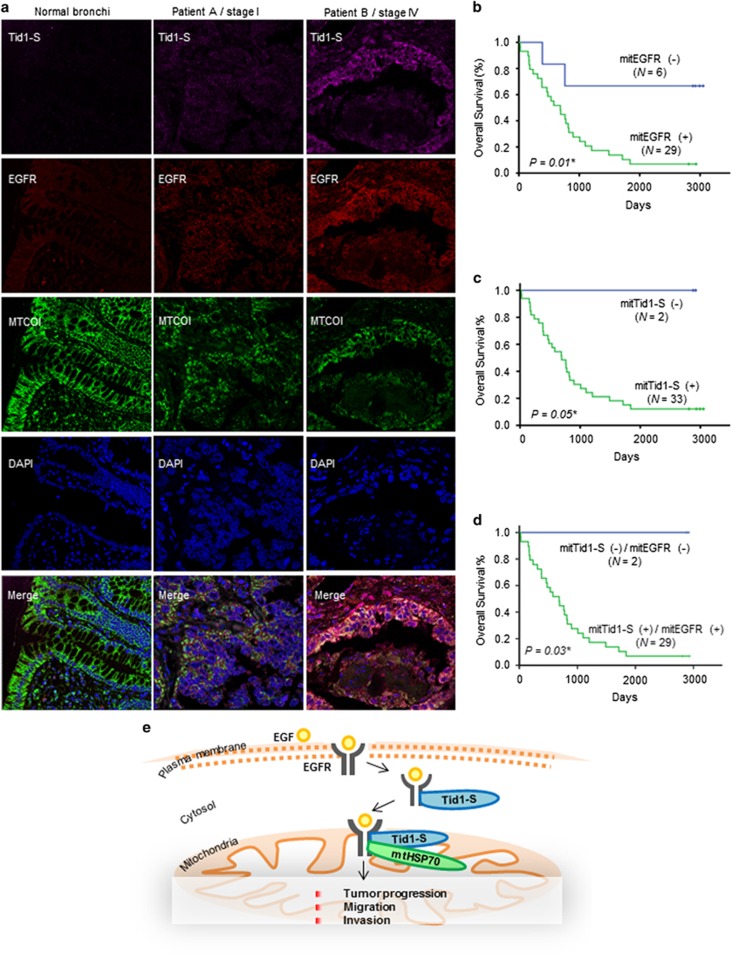
Expression levels of mitochondrial Tid1-S and EGFR in NSCLC tissues. (**a**) IF staining of Tid1-S, EGFR and MTCOI in NSCLC tissues. The subcellular distribution of Tid1-S, EGFR and MTCOI in normal bronchi (left column), tumor tissues from Stage I Patient A (middle column) and Stage IV Patient B (right column) were determined by IF staining. DAPI was used to counterstain nuclei. (**b**–**d**) Kaplan–Meier analysis of the overall survival of NSCLC patients stratified according to their mitEGFR score (**b**), mitTid1-S score (**c**), and representative multivariate data (**d**), group into mitTid1-S (−)/mitEGFR (−) or mitTid1-S (+)/mitEGFR (+). (**e**) A schematic model of how mitochondrial Tid1-S regulates the mitochondrial localization of EGFR in NSCLC. Tid1-S interacts with EGFR in the cytosol and translocates into mitochondria, where EGFR/Tid1-S/mtHSP70 complexes promote cell migration, invasion and tumor progression.

To evaluate the role of Tid1-S in mitochondrial EGFR accumulation and metastasis *in vivo*, we performed IF staining of EGFR, Tid1-S, and MTCOI in 35 paired tumor and adjacent normal tissues obtained from NSCLC patients of various stages. In normal bronchi of all patients, the mitochondria stained negative for Tid1-S and EGFR (Figure 5a, left panel). In cancerous lesions from overall stage I NSCLC patients, weak staining of EGFR and/or Tid1-S was detected in mitochondria (Figure 5a, middle panel/patient A). In cancerous lesions from overall stage IV NSCLC patients, strong staining of EGFR and Tid1-S was detected in mitochondria (Figure 5a, right panel/patient B). Significant correlations were found between high levels of mitTid1-S/mitEGFR and T stage (*P*=0.01), N stage (*P*=0.03), overall stage (*P<*0.01) and grade (*P*<0.01) (Table 1). We also observed a significant correlation between the expression levels of mitTid1-S and mitEGFR (*P*=0.01; Table 2). These results reveal that mitTid1-S/mitEGFR participates in the cancer growth and invasion of NSCLC *in vivo*.

To perform multivariate analysis of the mitochondrial expressions of Tid1-S and EGFR and their impacts on survival, we dichotomized the scores for MTCOI-associated Tid1-S (mitTid1-S) and EGFR (mitEGFR) into high (+) or low (−) expression as follows: mitTid1-S (+), intense staining of Tid1-S in mitochondria; mitTid1-S (−), little or no staining of Tid1-S in mitochondria; mitEGFR (+), intense staining of EGFR in mitochondria; and mitEGFR (−), little or no staining of EGFR in mitochondria. The levels of mitEGFR or mitTid1-S in the NSCLC (T) and adjacent normal (N) tissues of each patient were determined by quantitative IF analysis and expressed as the ratio of T/N in the histograms shown in Supplementary Figure 3. Our analysis revealed that patients with mitEGFR (−), mitTid1-S (−), or mitTid1-S (−)/EGFR (−) had better overall survival rates than those with mitEGFR (+), mitTid1-S (+), or mitTid1-S (+)/EGFR (+) (Figures 5b–d). These results suggest that high levels of Tid1-S/EGFR in mitochondria predict lymph node metastasis and poor overall survival in patients with NSCLC.

## Discussion

Two alternatively spliced forms of Tid1 that differ at their C-termini are expressed in human cells: Tid1-L and Tid1-S.^19^ Tid1 is synthesized as a cytosolic protein, and metalloproteases cleave the protein at consensus mitochondrial targeting sequences to allow it to be translocated into mitochondria. Tid1-L has a greater cytosolic stability and reduced rate of mitochondrial import compared with Tid1-S, resulting in higher levels of Tid1-L in the cytoplasm.^11^ Tid1-L, but not Tid1-S, interacts with EGFR/HSP70/HSP90 through the DnaJ domain, and this has been shown to counteract the ability of HSP90 to regulate EGFR by triggering the ubiquitinylation and proteasomal degradation of EGFR.^17^ In this work, we demonstrate that Tid1-S, but not Tid1-L, is involved in the EGF-stimulated translocation of EGFR into mitochondria in NSCLC cells. We further show that the DnaJ domain of Tid1-S interacts with mtHSP70, but not HSC70 (Figure 3), and appears to be indispensable for the translocation of EGFR into mitochondria (Figure 2). Therefore, depending on their subcellular localization and interaction with different chaperones, Tid1-L and Tid1-S may have very different actions in functions related to EGFR.

The subcellular localization of EGFR within mitochondria can affect aspects of cellular metabolism that are related to tumorigenesis. The translocation of EGFR into mitochondria has been shown to affect mitochondrial fission, energy production and metastasis in NSCLC.^6^ Moreover, the translocation of the related RTK, ErbB2, into mitochondria has been shown to affect the respiratory function of these organelles in breast cancer.^7^ In the present study, we demonstrate that overexpression of Tid1-S in cultured NSCLC cells enhances the mitochondrial accumulation of EGFR (Figure 2) and the migration and invasion abilities of these cells (Figure 4). Importantly, our analyses of the mitochondrial localization of Tid1-S and EGFR in clinical specimens from NSCLC patients reveal a very good correlation between high levels of mitochondrial Tid1-S/EGFR and lymph node metastasis and poor overall survival. However, our Kaplan–Meier analyses are likely to be skewed by very small number of the low expression cohorts. Inclusion of more NSCLC patients with low mitTid1-S and/or mitEGFR is needed to validate the significance of using low mitTid1-S/mitEGFR in predicting the overall survival of NSCLC patients.

In summary, we herein demonstrate that Tid1-S mediates the translocation of EGFR to mitochondria. We suggest that activated EGFR may undergo endocytosis and interact with Tid1-S to facilitate its transport to mitochondria, where the further interaction with mtHSP70 allows both EGFR and Tid1-S to undergo mitochondrial translocation (Figure 5e). Moreover, high levels of mitochondrial EGFR/Tid1-S promote metastasis-related activities in NSCLC. This metastasis-promoting function of Tid1-S is very different from the tumor-suppressor function of its alternatively spliced isoform, Tid1-L. In the future, studies examining how the alternative splicing of Tid1 is regulated should shed light on the tumorigenic roles of the alternatively spliced isoforms. Moreover, strategies aimed at modulating the relative expression levels of the two isoforms may prove clinically relevant in cancer therapeutics.

## Materials and methods

### Culture media, reagents and antibodies

Culture media and fetal bovine serum were purchased from Life Technologies (Grand Island, NY, USA). The antibodies against phospho-EGFR (Tyr1068) and E-cadherin (24E10) were purchased from Cell Signaling (Temecula, CA, USA). The antibodies against Tid1 (RS-13), Tid1-L (C-15), Tid1-S (S-9), EGFR (1005), lamin B (C-20) and MTCO1(C-20) were purchased from Santa Cruz Biotechnology, Inc. (Santa Cruz, CA, USA). The antibody against mitochondrial HSP70 (MA3-02) was purchased from Thermo Fisher Scientific, Inc. (Waltham, MA, USA). The antibody against HSC70 was purchased from Enzo Life Sciences, Inc. (Farmingdale, NY, USA). The antibody against calnexin (N-terminal region) was purchased from ECM Biosciences (Versailles, KY, USA). The antibody against β-actin was purchased from Sigma (St. Louis, MO, USA). The antibody against GAPDH (MAB374) was purchased from Merck Millipore Corporation (Billerica, MA, USA). EGF was purchased from R&D Systems, Inc. (MN, USA).

### Cell culture

The A549 human lung cancer cell line was purchased from the American Type Culture Collection (Manassas, VA, USA). The CL1-5 cell line was derived from CL1-0 cells using a Transwell invasion chamber, as previously described.^20^ CL1-5 cells were cultivated in RPMI-1640 containing 10% fetal bovine serum, 2 mm sodium pyruvate, 100 U/ml penicillin and 100 U/ml streptomycin. A549 cells were cultured in Dulbecco’s modified Eagle’s medium (DMEM) containing 10% fetal bovine serum, 0.5 mm sodium pyruvate, 2.5 mm l-glutamine, 100 U/ml penicillin and 100 U/ml streptomycin. All cell lines were confirmed to be free of mycoplasma contamination as tested by Mycoplasma PCR ELISA (Sigma). Cells were grown at 37 °C in a humidified incubator containing 5% CO_2_.

### Patients and tumor specimens

Tumor and adjacent normal tissue specimens were collected from 35 NSCLC patients who underwent surgical resection at Chang Gung Memorial Hospital (Tao-Yuan, Taiwan, ROC). Overall stage was grouped based on the status of primary tumor (T), regional lymph nodes (N) and distant metastasis (M). This study followed the tenets of the Declaration of Helsinki and was reviewed and approved by the Institutional Review Board and Ethics Committee of Chang Gung Memorial Hospital. Written informed consent was obtained from all patients.

### Plasmids and transfection

The plasmid for expression of wild-type Tid1-S-HA (pRK5-Tid1-S)^21^ was a gift from Jeng-Fan Lo (Institute of Oral Biology, National Yang-Ming University, Taipei, Taiwan, ROC) and is herein designated Tid1-S-Wt. Site-directed mutagenesis was used to generate a Tid1-S mutant in which residues H121P122D123 in the DnaJ domain were replaced with Q121N122A123.^15^ The full-length cDNA encoding the DnaJ domain mutant of Tid1-S was cloned into the *SalI* and *XhoI* sites of the pRK5-HA vector, and the resulting construct was designated Tid1-S-Mut. Transfection of plasmid DNA into cells was performed using Lipofectamine 2000 (Invitrogen, Carlsbad, CA, USA) according to the manufacturer’s protocol.

### RNA interference

Tid1 was downregulated by RNA interference-mediated inhibition of mRNA expression, using a mixture of four siRNAs (ON-TARGETplus SMARTpool; Dharmacon, Lafayette, CO, USA). The siGENOME nontargeting siRNA pool (Dharmacon) was used as the control. The four siRNAs targeting the human Tid1 mRNA (GenBank accession no. NM_002136) covered the following: nucleotides 1545-1563 from the start codon (A1-1: CGGAAACCUUGGUGUAGUU), nucleotides 1709-1727 (A1-2: GGGAAUGAAGCUUGUGUAU), nucleotides 746-764 (A1-3: CAACUUCGGUCGUGGAGGA) and nucleotides 1468-1486 (A1-4: UAGAAUUCCUUCAGGGUGA). Transfection was performed using the Dharmafect 1 transfection reagent (Dharmacon) according to the manufacturer’s instructions. In brief, exponentially growing cells were seeded in regular growth medium without antibiotics at 40–50% confluence. After 24 h, cells were transfected with 100 nm siRNA and then incubated for an additional 72 h.

### EGF treatment

Cells were grown in 150-mm dishes to 80% confluence and starved of serum. After 24 h, the serum-starved cells were treated with 50 ng/ml of EGF for 15 min.

### Subcellular fractionation

A Qproteome mitochondrial isolation kit (Qiagen, Venlo, Netherlands) was used for cytoplasmic and mitochondrial fractionation, according to the manufacturer’s protocol.

### Immunoprecipitation and western blotting

Immunoprecipitation and western blotting were performed as described previously.^15, 17^

### Migration and invasion assays

Migration and invasion assays were performed as described previously.^22^

### IF staining and analysis of colocalization

The distributions of Tid1-L, Tid1-S and EGFR in different locations of NSCLC tissues and cells were determined by IF staining as follows. Frozen NSCLC tissues were cut into 5 μm sections and adhered to glass slides, while NSCLC cells were placed on chamber slides. The slides were fixed with 4% paraformaldehyde in phosphate-buffered saline. The fixed cells were permeabilized with 0.1% Triton X-100, blocked with 3% bovine serum albumin for 1 h and incubated for 16 h with antibodies against Tid1-L (C-15), Tid1-S (S-9), EGFR (1005), or MTCO1 (C-20) (Santa Cruz Biotechnology). The samples were then incubated with Alexa Fluor 647 donkey anti-goat IgG (A21447), Alexa Fluor 594 donkey anti-rabbit IgG (A21207), or Alexa Fluor 488 goat anti-mouse IgG (A11001; all from Life Technologies-Molecular Probes).^6^ Tissues or cells were visualized under confocal microscopy (LSM 700; Carl Zeiss, Jena, Germany) and the results were processed using the Zen 2009 software (Carl Zeiss). The MetaMorph software (MetaMorph Inc., Nashville TN, USA) was used to analyze colocalization.^6^

### Statistical analysis

For nonparametric analysis, the Student’s *t*-test, Chi-square test and the Kaplan–Meier method were performed using Prism 5.0 (GraphPad Software, La Jolla, CA, USA) or the Statistical Package for the Social Sciences version 12.0 (SPSS Inc., Chicago, USA). Variances between two groups were analyzed with Student's *t*-test or Chi-squared test for clinical categorical variables. The overall survivals for clinical patients were assessed by the Kaplan–Meier method. Sample sizes were chosen based on pilot studies that certified acceptable statistical power with similar variances. Data were presented as means±s.d. Differences were considered significant at *P*<0.05. The researchers involved in the study were not completely blinded during sample collection or data analysis.

## Figures and Tables

**Table 1:** Correlations of high-level mitochondrial EGFR and Tid1-S with the pathological features of 35 NSCLC patients^a^

*Characteristics*	*Level of mitEGFR*			*Level of mitTid1-S*			*Levels of mitTid1-S and mitEGFR*		
	*Low*^b^ (N=*6)*	*High*^c^ (N=*29)*	P*-value*	*Low*^b^ (N=*2)*	*High*^c^ (N=*33)*	P*-value*	*Low*^b^ (N=*2)*	*High*^c^ (N=*29)*	P*-value*
*Age (years)*									
⩽60	3	6	0.13	1	8	0.42	1	7	0.41
>60	3	23		1	25		1	22	
									
*Gender*									
Male	3	12	0.70	2	13	0.09	1	12	0.81
Female	3	17		0	20		1	17	
									
*T stage*^d^									
T1	3	6	0.13	2	7	0.01*	2	6	0.01*
T2–T4	3	23		0	26		0	23	
									
*N stage*^e^									
*N*=0	4	8	0.06	2	10	0.04*	2	8	0.03*
*N*>0	2	21		0	23		0	21	
									
*M stage*^f^									
M=0	6	16	0.04*	2	20	0.26	2	16	0.21
M>0	0	13		0	13		0	13	
									
*Overall stage*									
I	4	5	0.01*	2	7	0.01*	2	5	<0.01**
II-/IV	2	24		0	26		0	24	
									
*Grade*^g^									
1	4	4	<0.01**	2	6	<0.01**	2	4	<0.01**
2–3	2	25		0	27		0	25	

a As assessed by *χ*^2^-test.

b Low: tumors showing little or no staining in mitochondria.

c High: tumors showing intense staining in mitochondria.

d T stage: the size and extent of the main tumor.

e N stage: the number of nearby lymph nodes that have cancer cells.

f M stage: whether the cancer has metastasized.

g Grade: to classify cancer cells based on their appearance and behavior when viewed under a microscope.

**Table 2:** Correlation between the level of mitTid1-S and mitEGFR in the tumors from 35 NSCLC patients

*Characteristics*	*Level of mitEGFR*		
	*Low*^a^ (N=*6)*	*High*^b^ (N=*29)*	P*-value*^c^
*Level of mitTid1-S*			
Low^d^ (*N*=2)	2	0	0.01*
High^e^ (*N*=33)	4	29	

a Low: tumors showing little or no staining of EGFR in mitochondria.

b High: tumors showing intense staining of EGFR in mitochondria.

c By *χ*^2^-test.

d Low: tumors showing little or no staining of Tid1-S in mitochondria.

e High: tumors showing intense staining of Tid1-S in mitochondria.
