# Supplementary figures and images for: Tid1-S regulates the mitochondrial localization of EGFR in non-small cell lung carcinoma
Source: Oncogenesis. 2017 Jul 17;6(7):e361–. doi: 10.1038/oncsis.2017.62 (PMC5541714; doi:10.1038/oncsis.2017.62)

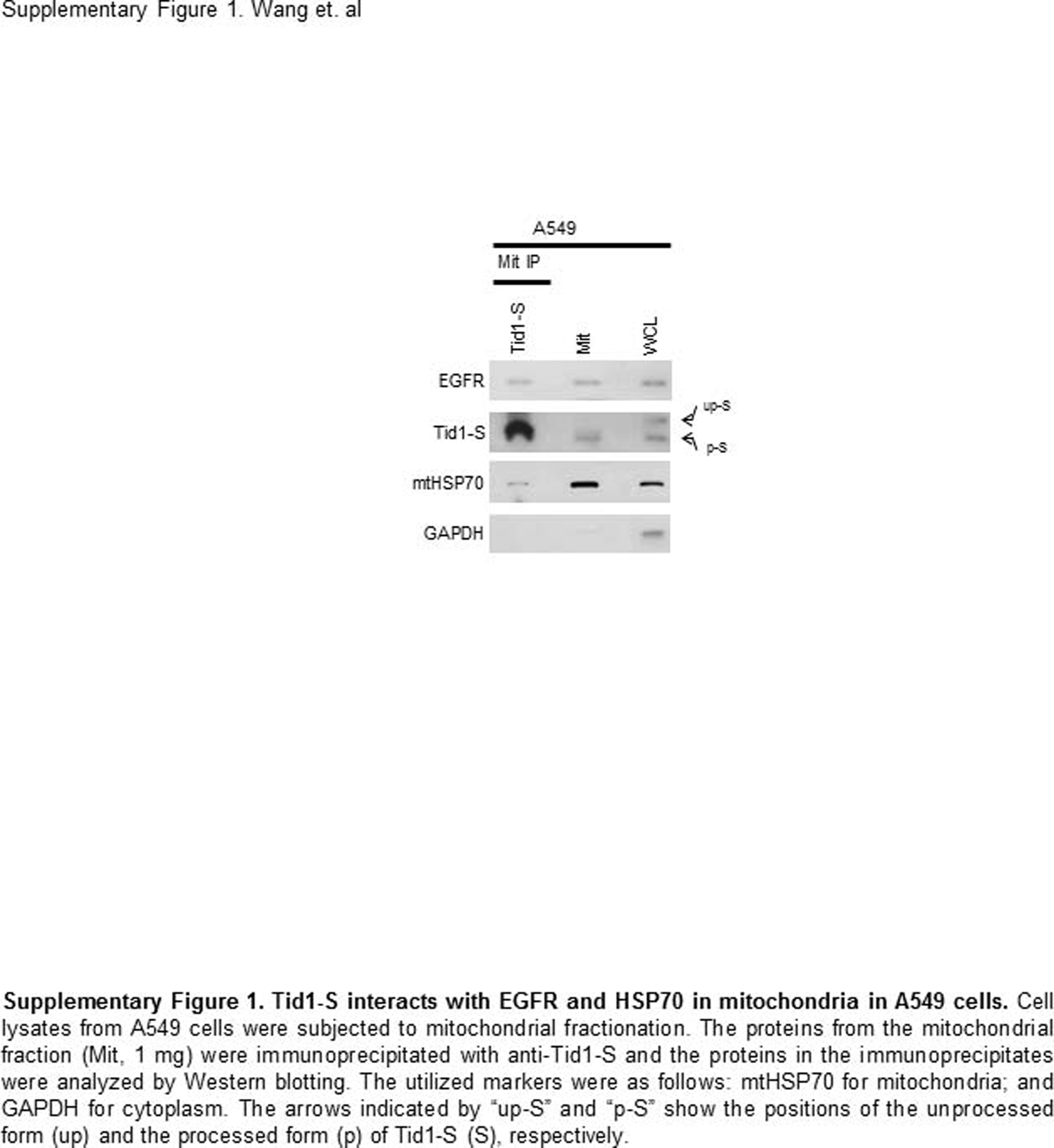

Supplement: Supplementary Figure 1 [file oncsis201762x1.tif]

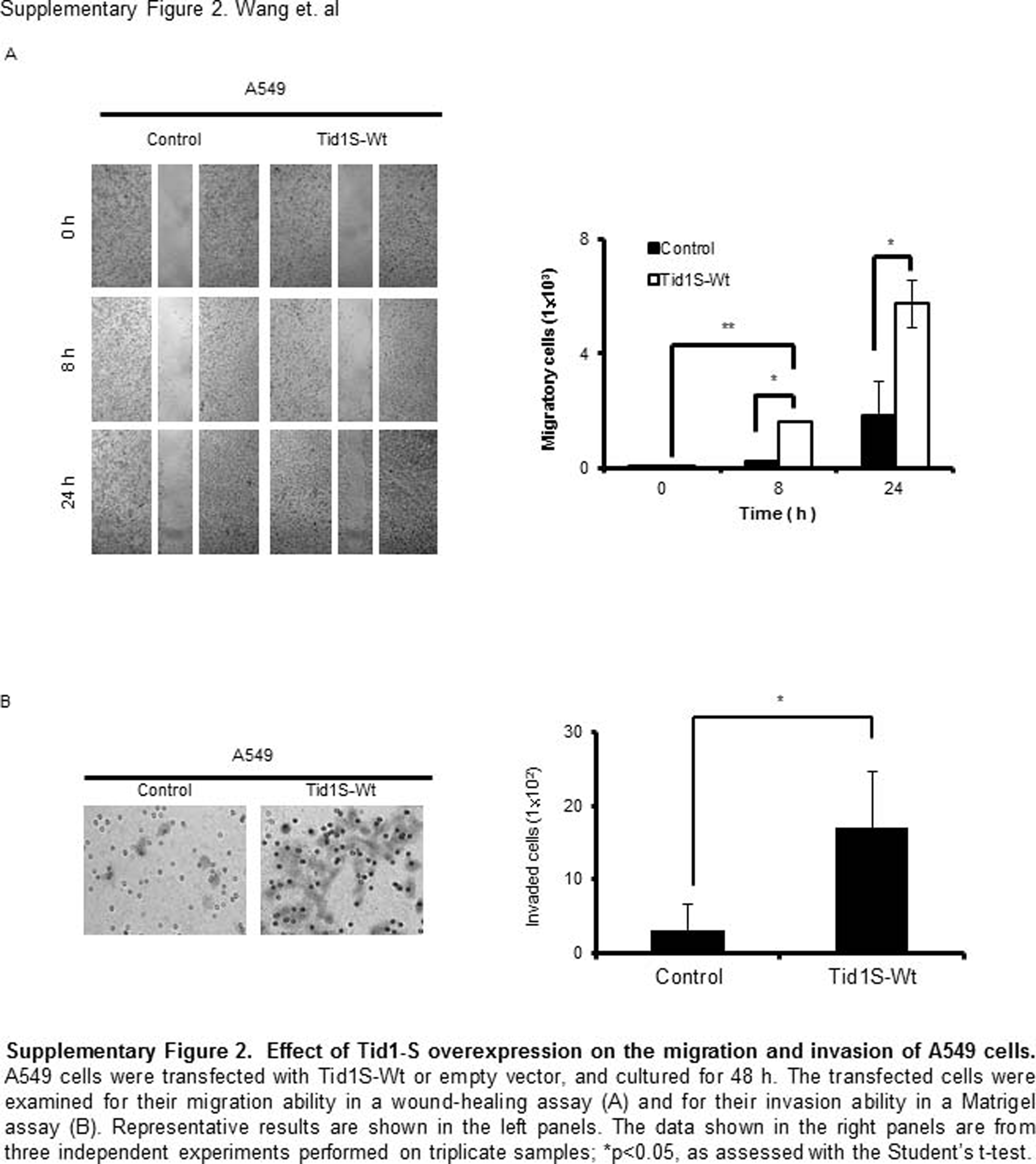

Supplement: Supplementary Figure 2 [file oncsis201762x2.tif]

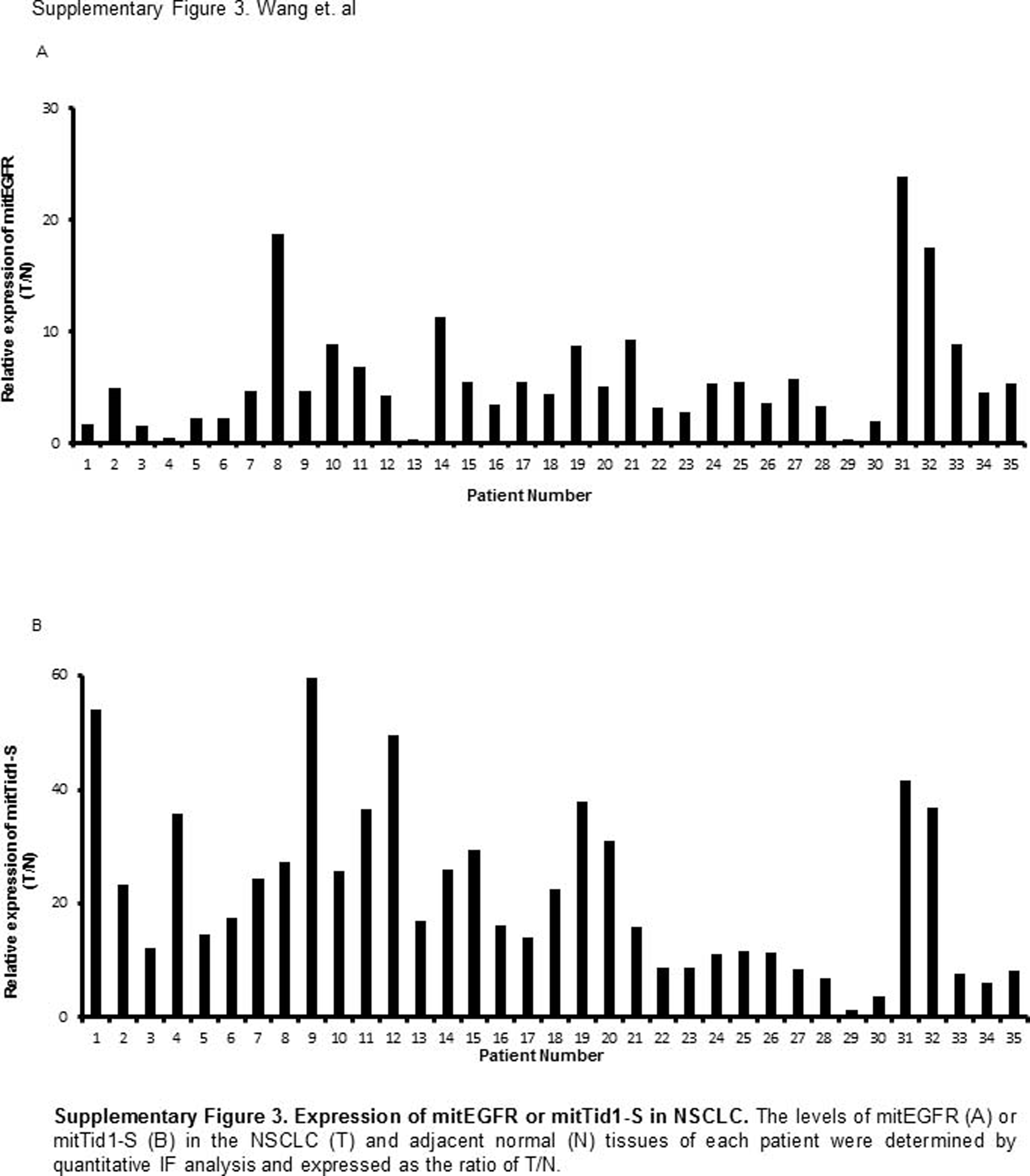

Supplement: Supplementary Figure 3 [file oncsis201762x3.tif]
